# Active steroid hormone synthesis renders adrenocortical cells highly susceptible to type II ferroptosis induction

**DOI:** 10.1038/s41419-020-2385-4

**Published:** 2020-03-17

**Authors:** Isabel Weigand, Jochen Schreiner, Florian Röhrig, Na Sun, Laura-Sophie Landwehr, Hanna Urlaub, Sabine Kendl, Katja Kiseljak-Vassiliades, Margaret E. Wierman, José Pedro Friedmann Angeli, Axel Walch, Silviu Sbiera, Martin Fassnacht, Matthias Kroiss

**Affiliations:** 1 Department of Internal Medicine I, Division of Endocrinology and Diabetes, University Hospital, University of Würzburg, Würzburg, Germany; 2 Department of Biochemistry and Molecular Biology, Theodor-Boveri-Institute, Biocenter, University of Würzburg, Würzburg, Germany; 3 Research Unit Analytical Pathology, Helmholtz Zentrum Munich, German Research Center for Environmental Health (GmbH), Oberschleißheim, Germany; 4 University of Colorado School of Medicine, Division of Endocrinology, Aurora, CO USA; 5 Research Service, Rocky Mountain Regional Veterans Affairs Medical Center, Aurora, CO USA; 6 Rudolf Virchow Center for Experimental Biomedicine, University of Würzburg, Würzburg, Germany; 7 Comprehensive Cancer Center Mainfranken, University of Würzburg, Würzburg, Germany; 8 Central Laboratory, University Hospital Würzburg, Würzburg, Germany

**Keywords:** Adrenal gland diseases, Adrenal tumours

## Abstract

Conditions of impaired adrenal function and tissue destruction, such as in Addison’s disease, and treatment resistance of adrenocortical carcinoma (ACC) necessitate improved understanding of the pathophysiology of adrenal cell death. Due to relevant oxidative processes in the adrenal cortex, our study investigated the role of ferroptosis, an iron-dependent cell death mechanism and found high adrenocortical expression of glutathione peroxidase 4 (GPX4) and long-chain-fatty-acid CoA ligase 4 (ACSL4) genes, key factors in the initiation of ferroptosis. By applying MALDI mass spectrometry imaging to normal and neoplastic adrenocortical tissue, we detected high abundance of arachidonic and adrenic acid, two long chain polyunsaturated fatty acids which undergo peroxidation during ferroptosis. In three available adrenal cortex cell models (H295R, CU-ACC1 and CU-ACC-2) a high susceptibility to GPX4 inhibition with RSL3 was documented with EC_50_ values of 5.7 × 10^−8^, 8.1 × 10^−7^ and 2.1 × 10^−8^ M, respectively, while all non-steroidogenic cells were significantly less sensitive. Complete block of GPX4 activity by RSL3 led to ferroptosis which was completely reversed in adrenal cortex cells by inhibition of steroidogenesis with ketoconazole but not by blocking the final step of cortisol synthesis with metyrapone. Mitotane, the only approved drug for ACC did not induce ferroptosis, despite strong induction of lipid peroxidation in ACC cells. Together, this report is the first to demonstrate extraordinary sensitivity of adrenal cortex cells to ferroptosis dependent on their active steroid synthetic pathways. Mitotane does not induce this form of cell death in ACC cells.

## Introduction

Cell death in the adrenal cortex is poorly understood but of high clinical relevance. In Addison’s disease, destruction of adrenocortical cells leads to a lack of adrenal steroids which—if untreated—may be fatal^1^. While it has become clear that polymorphisms of genes involved in the control of autoimmunity^2,3^ predispose to Addison’s disease, it is unclear how dying adrenocortical cells initiate antigen exposure that ultimately results in adrenal cortex destruction.

In contrast, uncontrolled proliferation of adrenocortical cells can result in neoplasms like adrenocortical carcinoma (ACC), a very rare malignancy with an overall poor prognosis^4,5^. Treatment options for ACC are scarce with mitotane (o,p′-DDD) being the only approved drug and used both for adjuvant treatment and in metastatic disease^6,7^. Adverse effects are frequent and often dose-limiting^5^. Nevertheless, objective response rates to mitotane alone or in combination chemotherapy are only approximately 20%^8,9^. These limitations fueled the search for novel and better treatment options against ACC; however with limited success to date^10,11^ (for review see^12^). The development of novel therapeutics is also hampered by the lack of knowledge about molecular mechanisms of mitotane action despite its specific adrenolytic activity^13^. Inhibition of mitochondrial respiration^14–16^ and sterol-o-acyl transferase (SOAT)1 have been shown to be involved^17^, and a SOAT1 inhibitor has been tested in a phase I clinical trial against ACC (NCT01898715).

Ferroptosis is an iron-dependent form of cell death associated with increased lipid peroxidation^18^, shown to be fully independent of caspase activity^19^ and pathophysiological roles for this cell death have been described in ischemic injuries such as renal failure^20,21^. Ferroptosis is tightly regulated by glutathione peroxidase 4 (GPX4)^22^ which belongs to the family of GPX enzymes that are able to reduce hydroperoxides at the expenses of two molecules of glutathione (GSH)^23^. Upon GPX4 inhibition lipid peroxidation is triggered which lead to the specific oxidation of an ill-characterized phosphatidylethanolamine (PE) pool^24^. Specifically, cells expressing Acyl-CoA synthase long-chain family member 4 (ACSL4)^25^ are particularly sensitive to ferroptosis^25^. ACSL4 preferentially catalyzes the esterification of arachidonic (ArA) and adrenic acid (AdrA) which are subsequently incorporated into phospholipids by the action of acyl transferases^24^. Ferroptosis can be pharmacologically induced by either depleting GSH levels, (so called type I inhibitors, such as erastin)^18,26^ or by blocking GPX4 activity, by type II inhibitors, such as (*1S,3R*)-RSL-3 (RSL3)^22,26^.

Given the relevance of oxidative processes in the adrenal gland and the pathophysiological importance of cell death in this critically relevant stress responsive organ, we here aimed to explore the role of ferroptosis in adrenocortical cells and its potential in future drug developments.

## Results

### Adrenocortical cells express ferroptosis-related proteins and accumulate adrenic and arachidonic acid

**Fig. 1:**
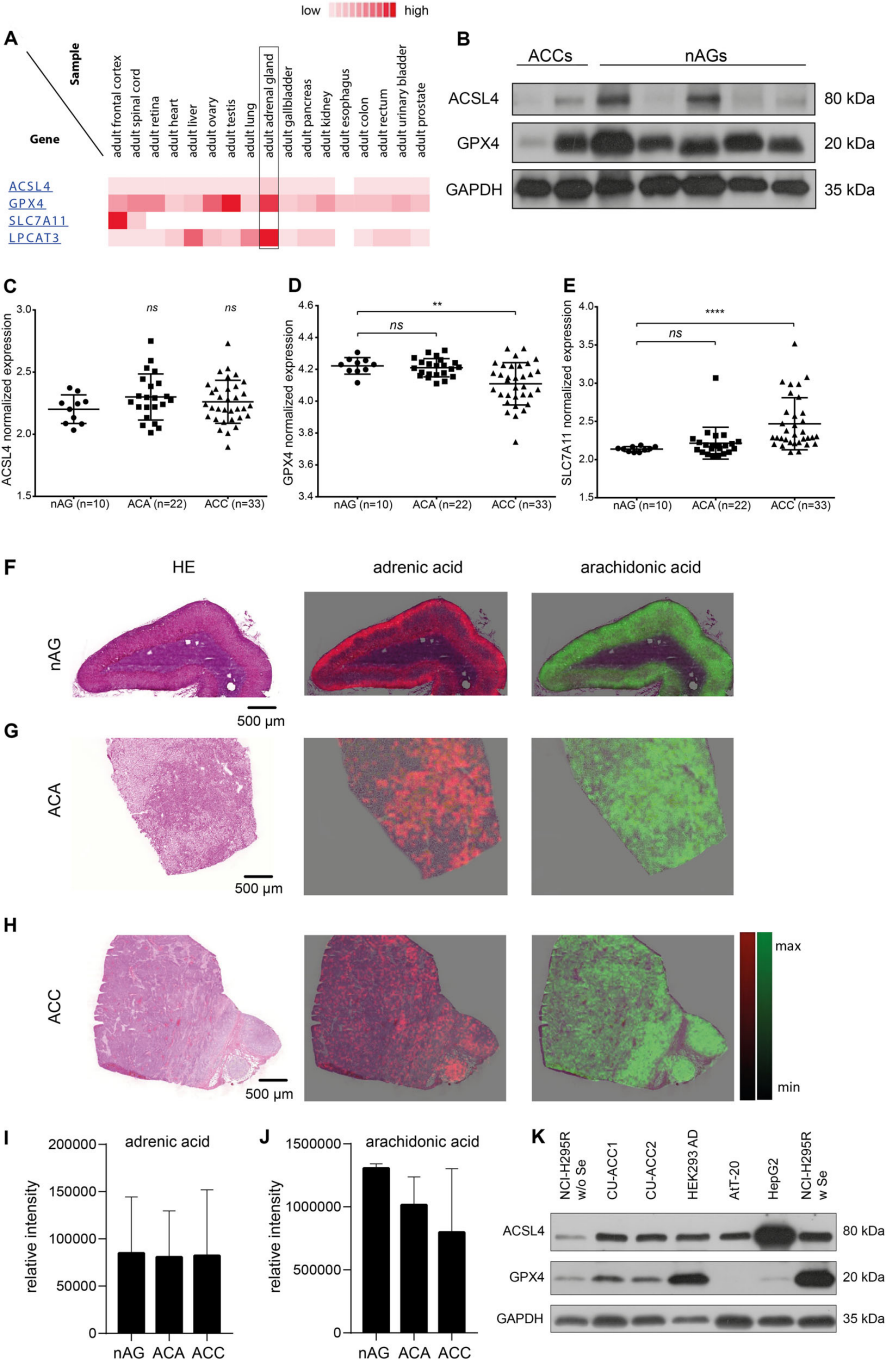
Expression of key ferroptosis macromolecules in the adrenal cortex. In silico expression analysis of four ferroptosis-related genes in the adult human adrenal gland, compared to other adult human tissues (**a**). Expression of ACSL4 and GPX4 in adrenocortical tissues detected by WB. nAG: normal adrenal gland, ACC adrenocortical carcinoma, α-tubulin served as loading control (**b**). In silico analysis and normalized expression of ACSL4 (**c**), GPX4 (**d**) and SLC7A11 (**e**) in normal adrenal glands (nAGs), adrenocortical adenomas (ACAs) and adrenocortical carcinomas (ACCs). Error bars represents standard deviations **p < 0.01, ****p < 0.0001. Hematoxylin/eosin stainings and MALDI-MSI intensity maps of adrenic acid (magenta) and arachidonic acid (green) in one representative nAG (**f**), ACA (**g**) and ACC (**h**). Relative intensities of adrenic acid (I) and arachidonic acid (**j**) did not differ between nAGs, ACAs and ACCs (one-way ANOVA). Expression of ACSL4 and GPX4 in three human ACC and three non-adrenal cell lines detected by WB. GAPDH served as loading control (**k**).

Adrenocortical steroid synthesis has been associated with an increased level of reactive oxygen species (ROS)^27^. We therefore hypothesized that adrenocortical cells might be inherently sensitive to ferroptosis via an increased basal level of lipid hydroperoxides. We initially investigated expression of genes involved in ferroptosis execution in adrenocortical cells, normal adrenal gland tissue and adrenocortical tumors. The HumanProteomeMap public database was mined and ferroptosis gene expression levels in the adult adrenal gland were compared to other tissues. GPX4 and lysophosphatidylcholine acyltransferase 3 (LPCAT3), an enzyme involved in incorporating polyunsaturated fatty acids (PUFAs) into membrane phopholipids, were clearly overexpressed in the adrenal cortex. In addition, we also found slightly higher expression of ACSL4 whereas SLC7A11 (a gene encoding one component of the cystine/glutamate antiporter System Xc^−^) expression was similarly low as in most other human tissues (Fig. 1a). We next analyzed the expression of the two most crucial ferroptosis regulating players, ACSL4 and GPX4, in five normal adrenal glands (nAGs) and two ACCs. ACSL4 expression varied in the two ACCs tested, with one showing only marginal expression and the other one showing a rather high expression (Fig. 1b). In the nAGs tested, ACSL4 expression varied as well with two out of three samples showing a high expression (Fig. 1b). In contrast, GPX4 protein was highly expressed in all adrenal samples tested, except one ACC (Fig. 1b). By analyzing publicly available microarray data^28^ of 10 nAGs, 22 adrenocortical adenomas (ACAs) and 33 ACCs, ACSL4 mRNA expression varied within all three groups without statistically significant differences (Fig. 1c). In contrast, GPX4 mRNA expression was similar in nAGs and ACAs, but was significantly reduced in ACCs (Fig. 1d). However, mRNA expression of SLC7A11 was significantly higher in ACCs compared to nAGs (Fig. 1e). Survival analyses of ACC patients in this data set was performed to test the impact of ferroptosis marker gene expression on patient outcome. In this small sample size only a low SLC7A11 expression, was associated with a trend towards better overall survival (hazard ratio, HR 2.3; 95% confidence interval, CI 0.9–6.1; *p* = 0.08) (Supplementary Fig. S1A-C).

Peroxidation of the two PUFAs AdrA and ArA is a hallmark of ferroptosis^24^. We, therefore determined the abundance of AdrA and ArA in fresh frozen tissue from each three nAGs, ACAs and ACCs by MALDI mass spectrometry imaging (MALDI-MSI), an innovative technology that permits the direct assessment of small molecules in tissue^29^. AdrA and ArA were both highly abundant in the cortex of nAG, but absent in the medulla (Fig. 1f). Similarly, high amounts of both AdrA and ArA were detectable in benign and malignant adrenocortical tumors (Fig. 1g–j).

We further analyzed the expression levels of ACSL4 and GPX4 in three adrenocortical cancer cell lines NCI-H295R^30^, CU-ACC1 and CU-ACC2^31^ which were subsequently used for all further experiments. All three ACC cell lines expressed both markers (Fig. 1k). GPX4 expression in the NCI-H295R cell line was extremely high in standard medium which contains selenium (Se) as a supplement (Fig. 1k). When cultured in Se-free medium, GPX4 expression in NCI-H295R cells was strongly reduced. Impaired cell viability led us to use NCI-H295R with Se supplementation as a model system for subsequent experiments.

### Adrenocortical cells are sensitive to ferroptosis induced by GPX4 inhibition

**Fig. 2:**
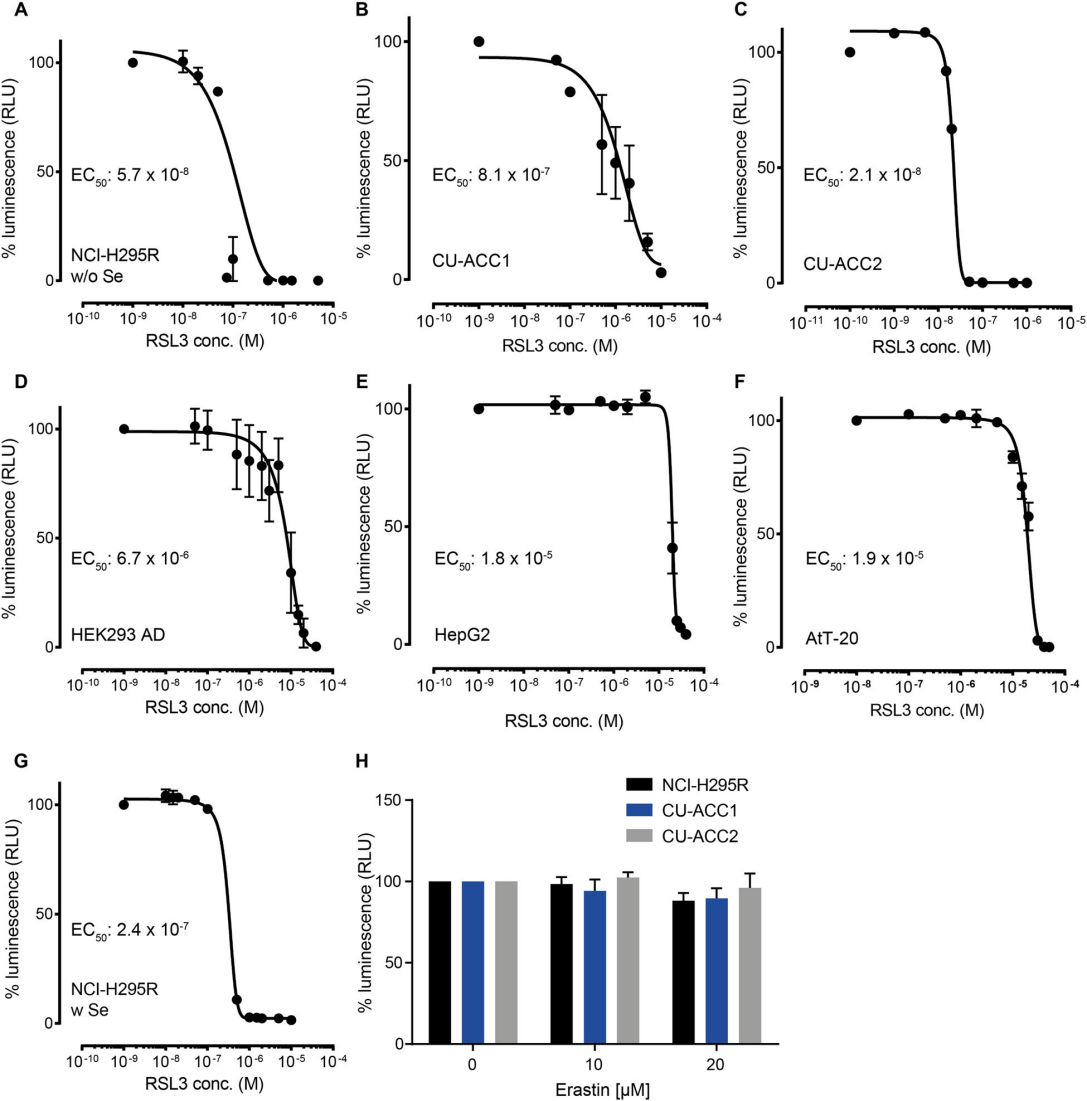
High susceptibility to type II ferroptosis of adrenocortical cells. Dose response curves (% luminescence (RLU), CellTiterGlo) of three human adrenocortical cell lines (**a**–**c** & **g**) (three independent experiments with eight biological replicates) and three non-adrenal cell lines (**d**–**f**) (two independent experiments with 8 biological replicates) after 24 h RSL3 treatment at increasing concentrations; error bars represent standard error of the mean. **h** Viability of three human adrenocortical cell lines after treatment with system Xc^−^ inhibitor erastin (10 µM and 20 µM) for 24 h in three human adrenocortical cell lines (three independent experiments with eight biological replicates); error bars represent standard error of the mean (**g**). Values normalized (100%) to vehicle-treated control.

Since ferroptosis-related proteins are highly expressed in cells and normal adrenal gland tissues, we tested the sensitivity of all adrenocortical cells to GPX4 inhibition in comparison to cells of non-adrenal origin by using various concentrations of RSL3 for 24 h. All three adrenocortical cell lines were highly susceptible to RSL3 treatment (Fig. 2a–c) with EC_50_ values of 5.7 × 10^−8^ M, 8.1 × 10^−7^ M and 2.1 × 10^−8^ M for NCI-H295R (w/o Se), CU-ACC1 and CU-ACC2, respectively. NCI-H295R cells cultured with Se were still highly, but less sensitive to RSL3 treatment (EC_50_: 2.4 × 10^−7^ M) (Fig. 2g). In contrast, cells of non-adrenal origin were at least one order of magnitude less sensitive to RSL3 treatment than adrenocortical cells (Fig. 2d–f) with EC_50_ values of 6.7 × 10^−6^ M, 1.8 × 10^−5^ M and 1.9 × 10^−5^ M for HEK-293AD, HepG2 and AtT-20 cells. We next investigated if the ferroptosis type I inducer, erastin, displays a similar potential to kill adrenocortical cell lines, but noted it lacked cytotoxicity in all three ACC cell lines (Fig. 2f).

### Inhibition of steroidogenesis reverses the cytotoxic effects of RSL3 in ACC cells

**Fig. 3:**
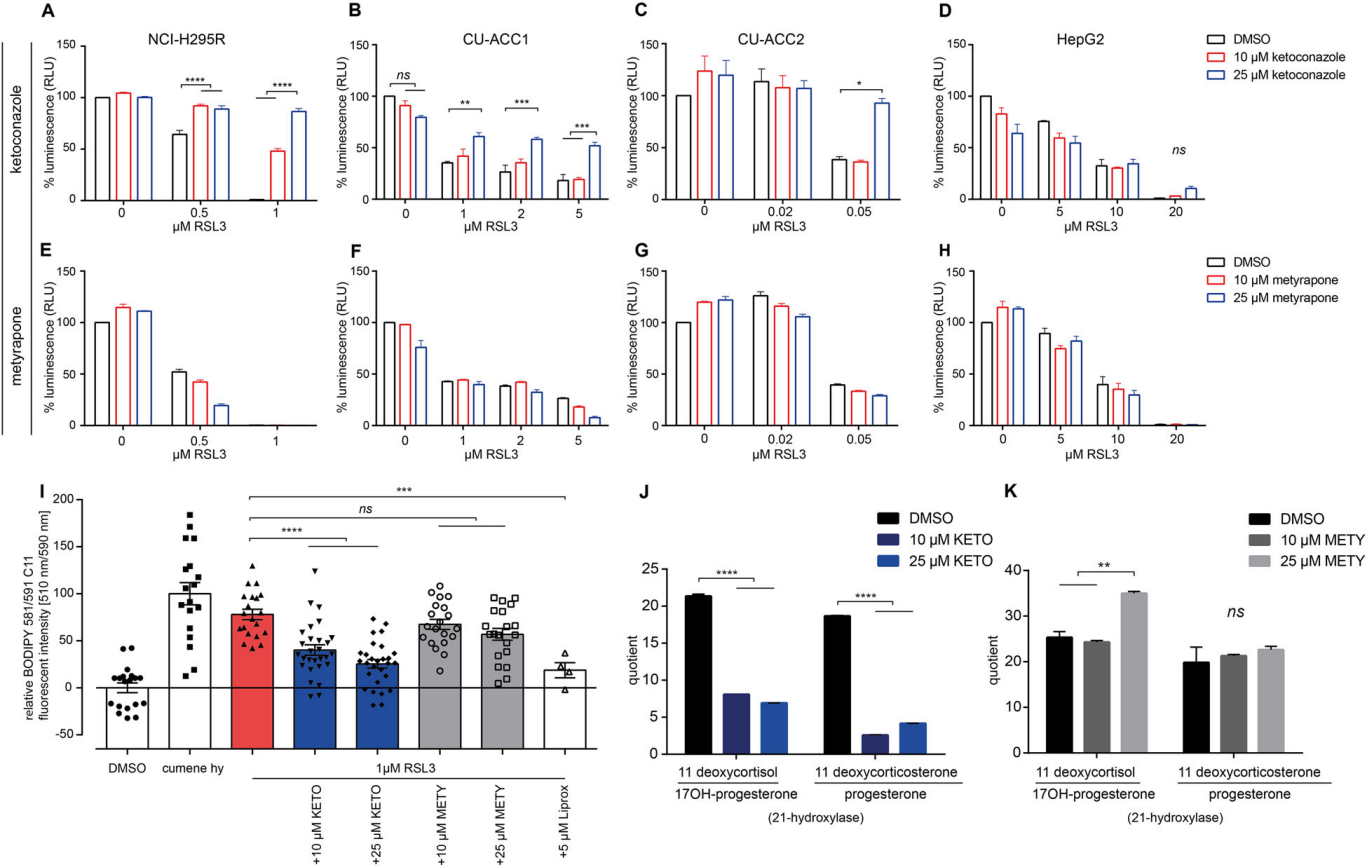
Upstream blockage of steroid synthesis overcomes GPX4 inhibition. Cell viability of NCI-H295R (**a**, **e**) (three independent experiments with eight biological replicates), CU-ACC1 (**b**, **f**), (three independent experiments with eight biological replicates) CU-ACC2 (**c**, **g**) (three independent experiments with eight biological replicates) and HepG2 cells (**d**, **h**) (two independent experiments with eight biological replicates) (CellTiterGlo) treated with RSL3 together with 10 µM and 25 µM ketoconazole (**a**–**d**) or 10 µM and 25 µM metyrapone (**f**–**h**), respectively; error bars represent standard error of the mean. Values normalized (100%) to vehicle-treated control. **i** Lipid peroxidation by BODIPY 581/591 C11 assay in NCI-H295R cells treated with RSL3 and different inhibitors of steroidogenesis. Normalization was performed by setting vehicle-treated control (DMSO) to 0 and positive control (Cumene hy) to 100%. **j** Quotient of 11 deoxycortisol/17OH-progesterone and 11 deoxycorticosterone/progesterone under ketoconazole and **k** metyrapone treatment. Groups were compared by one-way ANOVA, ****p < 0.0001, ***p < 0.001, **p < 0.01, *p < 0.05, ns=not significant. KETO: ketoconazole, METY: metyrapone, cumene hy: cumene hydroperoxide, Liprox: liproxstatin-1.

To test if blocking adrenal steroidogenesis might protect adrenocortical cells from the cytotoxic effects of type II ferroptosis, we treated NCI-H295R cells with RSL3 in combination with the clinically used pan-steroid inhibitor ketoconazole^32,33^. While ketoconazole at 10 and 25 µM single agent treatment (control) for 24 h had no effect on cell viability (Fig. 3a), steroid secretion was blocked as expected, e.g. androstendione secretion was reduced from 49.4 µg/l ± 1.7 to 26.2 µg/l ± 0.2 and 27.2 µg/l ± 0.5. For detailed results of all hormones measurable in both cell lines see Fig. S2A. Strikingly, RSL3 induced cell death was also rescued at doses of 10 and 25 µM (48 ± 4.4 and 87 ± 5.2% living cells, respectively (*p* < 0.0001)) (Fig. 3a). Similarly, 25 µM ketoconazole reverted RSL3 induced cell death in CU-ACC1 and CU-ACC2 cells (52 ± 5.6% (*p* < 0.001) and 92 ± 7.8% (*p* < 0.05), respectively) (Fig. 3b, c). Of note, ketoconazole did not significantly change the response of the non-adrenocortical HepG2 cells to RSL3 (Fig. 3d).

We next used metyrapone, a specific inhibitor of 11-β-hydroxylase, the enzyme catalyzing the last steps in cortisol (and aldosterone) synthesis. While metyrapone single agent (control) treatment blocked steroid secretion in the two steroidogenic ACC cells (NCI-H295R and CU-ACC1) at the utilized concentrations e.g., corticosterone in NCI-H295R cells was reduced from 2.82 µg/l ± 0.1 to 1.38 µg/l ± 0.01 and 1.3 µg/l ± 0.5 with 10 and 25 µM metyrapone, respectively (Supplementary Fig. S2C, D) it did not impair cell viability. At variance to ketoconazole treatment, the cytotoxic effects of RSL3 were not reverted in any of the ACC cell lines tested (Fig. 3e–h). In fact, when combined with intermediate doses of RSL3 (0.5 µM, for NCI-H295R and 0.02 µM for CU-ACC2) we observed reduced NCI-H295R and CU-ACC2 cell viability (Fig. 3e and g). Treatment with ketoconazole, but not metyrapone also significantly reduced RSL3-induced lipid peroxidation by two-fold (Fig. 3i). By calculating the ratio between 11-deoxycortisol and 17α-hydroxyprogesterone and 11-deoxycorticosterone and progesterone we found a significant reduction after ketoconazole treatment which could not be observed with metyrapone treatment (Fig. 3j, k). For an overview of steroid synthesis and steps blocked by different inhibitors, see Supplementary Fig. S3.

### Mitotane induces oxidation of lipids but not ferroptosis in adrenocortical cells

**Fig. 4:**
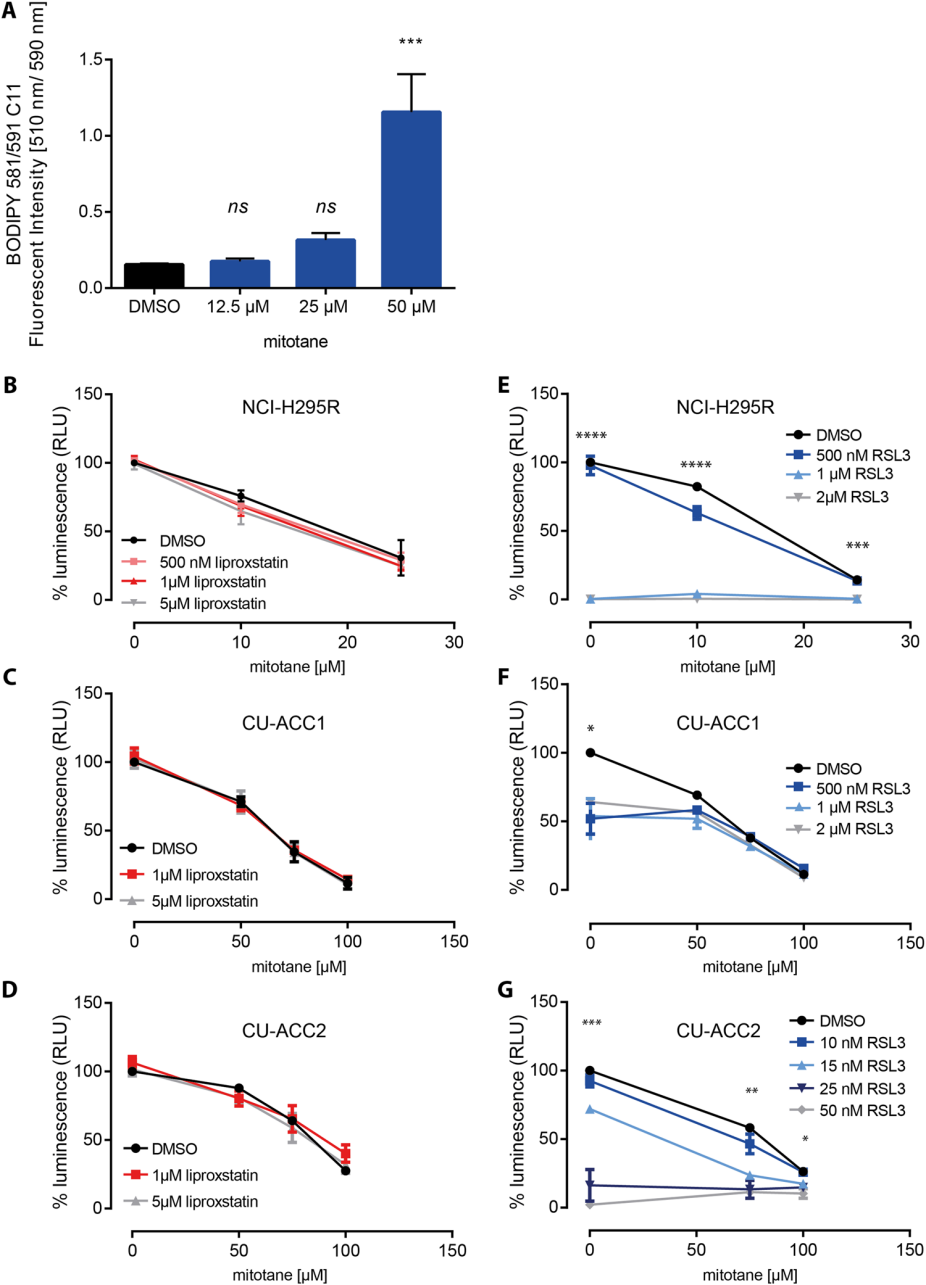
Mitotane induces lipid peroxidation but not ferroptosis. Lipid peroxidation by BODIPY 581/591 C11 is increased by mitotane treatment (**a**) (two independent experiments with eight biological replicates). The anti-ferroptotic molecule liproxstatin cannot rescue mitotane induced cell death in NCI-H295R cells (**b**), CU-ACC1 cells (**c**) and CU-ACC2 cells (**d**) (three independent experiments with eight biological replicates). RSL3 sensitizes all ACC cell lines to mitotane treatment (black line) (**e**–**g**) (three independent experiments with eight biological replicates) *p < 0.05 for difference between mitotane single agent and combination of the same mitotane concentration with RSL3. Values are normalized to control (=vehicle-) treated cells. Error bars represent standard error of the mean.

Mitotane is the only approved drug in ACC and exhibits relative selectivity for both normal and neoplastic adrenal cortex cells by poorly-defined molecular mechanisms. We hence investigated whether mitotane leads to the accumulation of oxidized lipids and induces ferroptosis in ACC cells. NCI-H295R cells showed increased lipid peroxidation in response to increasing concentrations of mitotane that doubled with 25 µM and increased by more than 7-fold with 50 µM mitotane treatment (*p* < 0.001) (Fig. 4a). To test whether ferroptosis plays a role in mitotane-induced cell death, cells were co-incubated with mitotane and the ferroptosis inhibitor liproxstatin-1. Different concentrations of liproxstatin-1 did not protect against the cytotoxic effects of mitotane in all three different human adrenocortical cancer cell lines (Fig. 4b–d). Since the GPX4 inhibitor RSL3 alone induced cell death in adrenocortical cells even at low concentrations (Fig. 2a–c), while erastin did not (Fig. 2g), we tested mitotane cytotoxicity in combination with RSL3 (Fig. 4e–g). In cells treated with 500 nM RSL3, 10 µM of mitotane reduced cell viability of NCI-H295R cells from 82 ± 1.5 to 63 ± 4.6% (*p* < 0.05) (Fig. 4e). Treatment with 25 µM of mitotane alone was sufficient to reduce cell viability to 14 ± 0.7% (Fig. 4e). In contrast, in CU-ACC1 cells, additional administration of RSL3 to mitotane had no significant effect on cell viability (Fig. 4f). However, in the extremely RSL3-sensitive CU-ACC2 cells, RSL3 led to a concentration dependent reduction of cell viability in combination with 75 µM mitotane (58 ± 2.5 to 46 ± 7.1% with 10 nM RSL3 and to 24% with 15 nM RSL3; *p* < 0.01; Fig. 4g).

## Discussion

**Fig. 5:**
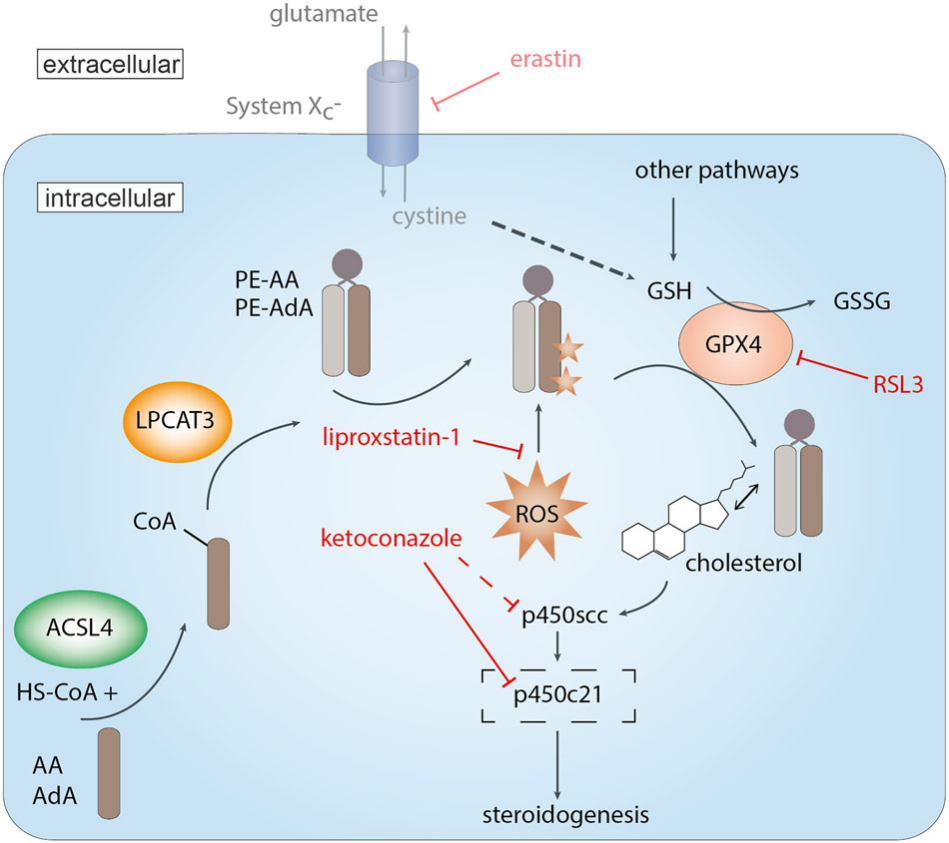
Model of ferroptosis and key molecules in the adrenal cortex. ACSL4 catalyzes activation of the PUFAs arachidonic acid (AA) and adrenic acid (AdA) which are incorporated by LPCAT3 in phospholipids, here mainly phosphatidylethanolamines (PE). Leaky cytochrome P450 activity of steroidogenesis leads to reactive oxygen species which peroxidize ArA and AdrA containing PE predominantly (star). GPX4 reduces peroxidized lipids at the expense of two molecules of glutathione (GSH) which is oxidized to glutathione disulfide (GSSG). GSH can be synthesized starting from cystine which is imported into many cells by system Xc^−^ which can be inhibited by erastin. In adrenocortical cells this system appears to play a minor role and other pathways are likely used for GSH synthesis. Interaction of cholesterol, the precursor of steroid hormones, with PUFA containing PE facilitates its entry into steroidogenesis by CYP11A1 (P450_scc_). Experimental blocking of steroidogenesis and very likely 21-hydroxylase with ketoconazole leads to decreased ROS production. GPX4 activity becomes less crucial for these cells and residual activity is sufficient to protect cells from ferroptosis.

In the present study, we for the first time demonstrate that the adrenal cortex disposes of critical enzymes and substrates required for ferroptosis. Inhibition of GPX4 mediated reduction of peroxidized PUFAs AdrA and ArA leads to type II ferroptosis. Active steroidogenesis is the potential cause for lipid peroxidation and susceptibility to ferroptosis in adrenal cortex cells. A model with key ferroptosis molecules investigated in this paper and a summary of our results is given in Fig. 5.

Adrenal cortical cells synthesize large amounts of steroid hormones from cholesterol and thus have an active machinery of cytochrome p450 (P450) oxidoreductases, enzymes using electrons from NADPH to reduce substrates. This system is highly “leaky” meaning not all electrons are equivalently transferred to target substrates, but to e.g., O_2_ instead^34^. Since most steps during steroidogenesis depend on different p450 enzymes, steroidogenesis contribute significantly to ROS production in the adrenal gland. High ROS production forces these cells permanently to face oxidative stress and for this reason, the adrenal gland is well equipped with several enzymatic and non-enzymatic antioxidant mechanisms^27,35^. PUFAs play a crucial role as stimulators of steroidogenesis^36^ e.g., by facilitating cholesterol binding to processing enzymes^37^. Thus, enrichment of AdrA in PEs from rat adrenal mitochondria was shown to facilitate cholesterol cleavage into pregnenolone by CYP11A1 (P450 side-chain cleavage, P450scc)^37^. We here demonstrated high abundance of both PUFAs in nAGs and benign and malignant adrenocortical tumors. Interestingly, in the non-steroidogenic adrenal medulla, both PUFAs were not detected by MALDI-MSI (Fig. 1). This underscores a physiological relevance of high AdrA and ArA levels in the adrenal cortex during steroidogenesis. It has been shown that primarily peroxidation of PEs containing the two PUFAs ArA and AdrA result in ferroptosis in mouse embryonic fibroblasts^24^.

Other factors predicting sensitivity to ferroptosis are ACSL4 and GPX4 expression^25^ although Yang et al. found reduced GPX4 expression to even sensitize cells to ferroptosis^22^. ACSL4 expression was already demonstrated to be high in mouse adrenal glands^38^ and the strong expression of GPX4 in all adrenal tissues supports a high demand for reducing peroxidized lipids within this tissue (Fig. 1b).

RSL3 is a potent inhibitor of GPX4^20^ and since GPX4 is the only enzyme that reduces esterified peroxidized fatty acids and cholesterol hydroperoxides^39^, inhibition of this enzyme results in the accumulation of peroxidized lipids and subsequently ferroptosis. Since NCI-H295R culture medium is supplemented with a mixture of insulin, transferrin and selenium, we tested if this affects GPX4 expression and sensitivity to RSL3. By culturing NCI-H295R cells in CU-ACC medium that does not contain this supplement, we observed a striking reduction of GPX4 expression (Fig. 1k) and an increased sensitivity to RSL3 treatment (Fig. 2g), which is in line with a very recently published study^40^. NCI-H295R cells without Se, although more sensitive to RSL3, were still not as sensitive as CU-ACC2 cells (Fig. 2). The low GPX4 expression in CU-ACC2 cells, in addition to the rather high expression of ACSL4 might explain the sensitivity of this cell line to RSL3 (Fig. 1k). Two independent studies published after the submission of this manuscript demonstrated ferroptosis-suppressor protein 1 (FSP1) as a novel protective mechanism against ferroptosis independently of GPX4 and GSH. The authors found that expression of FSP1 predicts sensitivity to RSL3^41,42^. How FSP1 expression contributes to RSL3 sensitivity in adrenocortical cells, needs to be elucidated in the future. Interestingly, erastin, which inhibits System Xc^−^, the antiporter responsible for cystine uptake into cells^43^ did not affect cell viability in any of the ACC cells (Fig. 1e). Since GPX4 is dependent on GSH, inhibition of cystine uptake theoretically leads to reduced intracellular GSH levels, reduced GPX4 activity and hence, increased ferroptosis. It has been previously demonstrated in other cell types that erastin treatment may lead to increased proliferation^44^. Accordingly, different cell types dispose of alternative mechanisms to compensate reduced cystine and GSH levels^45^, e.g, by transsulfuration, in which methionine is converted into cysteine^46^. Although adrenocortical cells are not dependent on cystine uptake (as suggested by the results from erastin treatment), they are dependent on proper GPX4 activity. This suggests that in ACC, cysteine may be generated from methionine through transsulfuration pathways which might compensate reduced levels of cystine.

Inhibition of CYP11A1 and other steroidogenic enzymes with ketoconazole, but not inhibition of CYP11B by metyrapone^32,33^, largely reversed RSL3 induced cell death indicating that only near complete inhibition of steroidogenesis is able to rescue cells from type II ferroptosis. The early block of steroidogenesis by ketoconazole results in fewer peroxidized lipids (Fig. 3i) and thus renders these cells less sensitive to GPX4 inhibition. Since the protective effects of ketoconazole were not observed in the non-steroidogenic HepG2 cell line, steroidogenesis thus gives an explanation why adrenocortical cells are particularly sensitive to ferroptosis induction. Surprisingly, early block of steroidogenesis also reversed ferroptosis induced by RSL3 in the non-steroidogenic CU-ACC2 cell line, supporting the idea that this cell line has the characteristics of steroidogenic cell lines despite unmeasurable steroid production. This is supported by high expression of CYP11A1 in CU-ACC2 cells (Supplementary Fig. S2E). One could argue that ketoconazole also may affect cholesterol synthesis by inhibiting CYP51^47^. We could exclude this possibility since inhibition of cholesterol synthesis with Tasin-1 did not alter susceptibility to RSL3 inhibition (Supplementary Fig. S5A-D). It has been further demonstrated that ketoconazole is able to reduce lipid peroxidation^48^ and might inhibit NADPH oxidase^49^ which possibly contributes to ferroptosis suppression in at least some cell lines after erastin treatment^18^. To limit the possibility of non-steroidogenic effects to be responsible for the anti-ferroptotic effect of ketoconazole, we treated the NCI-H295R cell line with the pan-steroidogenesis inhibitor etomidate^50^ which significantly rescued from RSL3-induced toxicity, although to a lesser extent (Supplementary Fig. S4A). On the other hand, RSL3-induced cell death in non-steroidogenic HepG2 cells was partially rescued by ferrostatin and liproxstatin (Supplementary Fig. S4F, G) but not ketoconazole (Fig. 3d) which further supports a specific effect through steroid synthesis inhibition.

No narrow down the actual steroidogenic enzyme(s) that may be responsible for susceptibility to ferroptosis we used inhibitors of specific steps in steroidogenesis (aminoglutethimide: CYP11A1; abiraterone and galeterone: CYP17)^50,51^ which however could not rescue from RSL3-induced toxicity (Supplementary Fig. S4B-D). We assumed that 21-hydroxylase might be the enzyme responsible for RSL3 sensitivity. To test this hypothesis, we made use of LC-MS/MS steroids hormone panel analysis in cell culture supernatants and found the diagnostic ratios 11 deoxycorticosterone/progesterone and 11 deoxycortisol/17-OH progesterone indicative of CYP21 activity to be decreased with ketoconazole (Fig. 3i) but not metyrapone treatment (Fig. 3j). Although without a specific CYP21 inhibitor available we cannot rule out effects beyond steroidogenesis to possibly contribute to the action of ketoconazole, our data strongly indicate a tissue specific requirement of anti-ferroptotic mechanisms in the adrenal cortex. In addition, no increased sensitivity towards RSL3 was observed when NCI-H295R cells were incubated with different steroid precursors (Supplementary Fig. S4E).

It is well established, that during ferroptosis lipid hydroperoxides accumulate^52^. Lipid hydroperoxides are either generated from the cytoplasmic labile iron pool (Fe^2+^) via the Fenton reaction^53^, a spontaneous, iron-catalyzed peroxyl radical-mediated process, called autoxidation^54^, or enzyme-mediated by Fe-dependent lipoxygenases^55,56^. Although mitotane exposure induced lipid peroxidation it did not result in ferroptosis since liproxstatin-1 could not reverse its cytotoxic effects. Liproxstatin-1, as ferrostatin-1, is a compound originally identified in a high-throughput screen as ferroptosis inhibitor^20^ able to act as a radical-trapping antioxidant, rather than inhibiting lipoxygenases^57^. In contrast, mitotane is known to affect mitochondrial respiration^14^ which is the major source for ROS within all cells. Increased lipid peroxidation under mitotane treatment is therefore likely, although independent of ferroptosis. While this paper was under review, another study was published demonstrating ferroptosis-independent cell death induced by mitotane^40^.

Our results demonstrate that cells of the adrenal cortex, due to its steroidogenic nature, are particularly sensitive to type II ferroptosis induction. These cells are well equipped with protective mechanisms against oxidative stress and contain both enzymes and lipids that are crucial for ferroptosis execution.

These mechanisms are overcome in severe conditions such as in adrenal insufficiency or sepsis. On the other hand, ferroptosis inducing agents might be a promising treatment option for ACC in the future as induction of ferroptosis has been investigated as a treatment of other malignancies^58,59^. Although RSL3 has been tested in vivo effectively without severe toxicity^22^ it is not properly suitable for in vivo use due to low solubility^60^. Following our results, a more stable and in vivo-use-suitable form of erastin, imidazole ketone erastin^59^, likely does not present a promising treatment option for ACC, either. Limited treatment options against ACC, with the only drug available, mitotane, not inducing ferroptosis, the development of novel and stable ferroptosis inducers or drugs that interfere with alternative pathways of glutathione synthesis might be a very promising treatment approach against ACC in the future.

## Materials and methods

### Cell culture

NCI-H295R cells were obtained from ATCC and cultured in DMEM/F12 supplemented with 1x Insulin-Transferrin-Selenium and Nu-Serum (2.5 %) (for culturing conditions with Se supplementation). CU-ACC1 and CU-ACC2 cells were obtained by KKV and MEW and cultured as described^31^. In brief, a 1:3 mixture of F12 Ham and DMEM high glucose (both Gibco) was supplemented with 10% FCS, 0.4 µg/ml hydrocortisone (Sigma-Aldrich), 5 µg/ml insulin (Sigma-Aldrich), 8.4 ng/ml cholera toxin (Sigma-Aldrich), 24 µg/ml adenine (Sigma-Aldrich) and 10 ng/ml EGF (Invitrogen). NCI-H295R cells without Se supplementation were also cultured in this media. Non-adrenal cell lines were cultured in DMEM (Gibco) supplemented with 10% FCS. STR analysis confirmed cell lines and cells were checked regularly for mycoplasma contamination. Mitotane was purchased from ISP Columbus and solved in EtOH except for lipid peroxidation assays. For this purpose, mitotane was solved in DMSO. Liproxstatin and ferrostatin were purchased from Sigma-Aldrich, erastin and RSL3 from Selleckchem. Ketoconazole and metyrapone were purchased from Sigma-Aldrich. If not stated otherwise, cells were treated with drugs for 24 h and compounds inhibiting any form of cell death were added 1 h before drug treatment initiated.

### Cell viability assays

To test effects of drugs on cell viability, CellTiter Glo Assay (Promega) was used according to the manufacturer’s protocol. 5 × 10^4^ cells were seeded in black 96 well plates with clear bottom 24 h prior to treatment. Equal amounts of CellTiterGlo 3D reagent were added to the media, mixed, incubated at RT for 25 min and the assay was measured on a Wallac Victor multilable plate reader. Exact number of replicates is indicated in each figure.

### SDS-PAGE and WB

Cells were lysed in RIPA Buffer (Sigma-Aldrich) containing protease inhibitor (Sigma-Aldrich) and phosphatase inhibitor (Santa Cruz) cocktails. 10 µg protein were loaded on a 4–15% denaturing gradient gel and proteins were separated by SDS-PAGE. Proteins were transferred by tank blot onto a nitrocellulose membrane that was subsequently blocked in 5 % skimmed milk in TBS-Tween at RT for 1 h. Primary antibodies (GPX4: Abcam #ab41787, 1:1000; ACSL4: Santa Cruz #sc271800, 1:1000; cleaved caspase3: Cell Signaling #9661 S 1:150, CYP11A1: abcam #ab75497 1:500, α-tubulin: Sigma #T9026 1:20000, GAPDH: Sigma #g9549 1:10000) were incubated over night at 4 °C. Membranes were washed three times in TBS-Tween and HRP-labeled secondary antibodies (goat-anti rabbit: Jackson ImmunoResearch Laboratories, #111–035–144 and goat-anti mouse: Jackson ImmunoResearch Laboratories, #115–035–003) were diluted 1:10000 and incubated at RT for 1 h. The protein-antibody complex was visualized by enhanced chemiluminescence using Amersham ECL Prime reagent (GE Healthcare) and documented on X-ray film (Fuji).

### Lipid peroxidation assay

Lipid peroxidation was detected with the Image-iT Lipid peroxidation kit (Life Technologies, #C10445). NCI-H295R cells were seeded in black 96-well plates with clear bottom and treated with mitotane, RSL3, ketoconazole and metyrapone for 24 h. 10 µM of sensor were added to the cultures during the last 30 min of incubation. Cells were washed thrice with DPBS and imaged immediately with a high throughput fluorescence microscope (Perkin Elmer, Operetta) with filters for FITC (Alexa488) and Texas Red (Alexa594). To quantify lipid peroxidation, signal intensities at 510 nm and 590 nm were used.

### Tissue collection

Fresh tumor samples from patients who underwent surgery for adrenal tumors were collected as part of the European Network for the Study of Adrenal Tumors (ENSAT) registry and biobank (https://registry.ensat.org) which was approved by the ethics committee at the University of Würzburg (approval # 86/03 and 88/11) and all patients provided informed consent. Anonymized normal adrenal glands were collected from patients who underwent adrenalectomy due to non-adrenal tumors. After surgery, fat and connective tissue were removed and tissues were immediately snap-frozen in liquid nitrogen and stored at −80 °C until proteins were extracted.

### MALDI imaging

Targeted quantification of arachidonic and adrenic acid in nAGs, ACAs and ACCs of fresh frozen tissues was performed as described elsewhere^29^.

### In silico analyses

An exploratory comparison of protein expression levels of ACSL4, GPX4, SLC7A11 and LPCAT3 in 17 normal adult human tissues was performed using the HumanProteomeMap (www.humanproteomemap.org) website that reorganizes mass spectrometry-based proteomics data deposited to the ProteomeXchange Consortium (http://www.proteomexchange.org) to explore expressed proteins in human tissues.

log2 normalized expression data (Human Genome U133 Plus 2.0 Kit, Affymetrix) of ACSL4 (probe 1557418_at), GPX4 (probe 201106_at) and SLC7A11 (probe 217678_at) in 10 normal adrenal glands (nAG), 22 adrenocortical adenomas, and 33 ACC^28^ were retrieved together with limited clinical data on the samples from the National Center for Biotechnology Information’s Gene Expression Omnibus (GSE10927). The normalized expression levels of individual samples have been plotted on the Y axis and the significance of the differences in expression levels have been analyzed by Kruskal-Wallis Test followed by Dunn’s multiple comparison test. For the adrenocortical carcinoma samples where survival data was available a Kaplan-Maier representation was performed using the median expression for each of the three proteins as cut-off value. A Long-rank (Mantel-Cox) test was performed for determining significance. A *P*-value under 5% was considered significant.

### LC-MS/MS of steroid hormones

Steroid hormones in cell culture supernatants of NCI-H295R and CU-ACC1 cells were quantified with the MassChrom steroids kit (Chromsystems) on a Qtrap 6500+ (Sciex) mass spectrometer coupled to a 1290 Infinity HPLC System (Agilent). Signal analysis was performed with Analyst Software (1.6.3, Sciex) as described elsewhere^61^.

### Statistical analysis

All experiments were performed independently with at least two, in general three biological replicates. If not indicated otherwise, statistical significance between different treatments was calculated using one-way ANOVA. To test equality of variance, Bartlett’s test was used. *P*-values below 0.05 were considered statistically significant and are indicated as follows: *p < 0.05, **p < 0.01, ***p < 0.001, ****p < 0.0001, ns = not significant. Statistical analyses were performed with GraphPad Prism 6.01.

## Supplementary information

Supplementary information

supplementary figure 1

supplementary figure 2

supplementary figure 3

supplementary figure 4

supplementary figure 5
