# Supplementary material for: Active steroid hormone synthesis renders adrenocortical cells highly susceptible to type II ferroptosis induction
Source: Cell Death Dis. 2020 Mar 17;11(3):192. doi: 10.1038/s41419-020-2385-4 (PMC7078189; doi:10.1038/s41419-020-2385-4)
Supplement: Supplementary file 1 — Supplementary information [file 41419_2020_2385_MOESM1_ESM.docx]

**Supplementary Information**

Active steroid hormone synthesis renders adrenocortical cells highly susceptible to type II ferroptosis induction

Isabel Weigand^1^*, Jochen Schreiner^1^*, Florian Röhrig^2^, Na Sun^3^, Laura-Sophie Landwehr^1^, Hanna Urlaub^1^, Sabine Kendl^1^, Katja Kiseljak-Vassiliades^4,5^, Margaret E. Wierman^4,5^, José Pedro Friedmann Angeli^6^, Axel Walch^3^, Silviu Sbiera^1^, Martin Fassnacht^1,7,8^ & Matthias Kroiss^1,7^

^1^ Department of Internal Medicine I, Division of Endocrinology and Diabetes, University Hospital, University of Würzburg, Würzburg, Germany

^2^ Department of Biochemistry and Molecular Biology, Theodor-Boveri-Institute, Biocenter, University of Würzburg, Würzburg, Germany

^3^ Research Unit Analytical Pathology, Helmholtz Zentrum Munich, German Research Center for Environmental Health (GmbH)

^4^ University of Colorado School of Medicine, Division of Endocrinology, Aurora, CO, USA

^5^ Research Service Veterans Affairs Medical Center, Rocky Mountain Regional Veterans Affairs Medical Center, Aurora, CO, USA

^6^ Rudolf Virchow Center for Experimental Biomedicine, University of Würzburg, Würzburg, Germany

^7^ Comprehensive Cancer Center Mainfranken, University of Würzburg, Würzburg, Germany

^8^ Central Laboratory, University Hospital Würzburg, Würzburg, Germany

*These authors contributed equally

**Supplementary Fig. 1**

Kaplan Meier plots of overall survival in in ACC patients of the TCGA cohort stratified by expression of ACSL4 (A), GPX4 (B) and SLC7A11 (C).

**Supplementary Fig. 2**

Steroid secretion of NCI-H295R (A) and CU-ACC1 (B) cells after ketoconazole treatment for 24 h and of NCI-H295R (C) and CU-ACC1 (D) cells after metyrapone treatment for 24 h. CYP11A1 expression of different cell lines (E). Only steroids measurable in the supernatants of both cell lines are shown. Note that cells were cultured for 24 h before inhibitors were added for another 24 h.

**Supplementary Fig. 3**

Overview of steroid synthesis in adrenocortical cells. Color coding: green: glucocorticoids and precursors, blue: mineralcorticoids, magenta: androgens and brown: estrogens. Only enzymes and genes mentioned in the manuscript are depicted. Main steps inhibited by ketoconazole (red) and metyrapone (yellow) are indicated^33^. In black, known enzymes inhibited by aminoglutethimide, etomidate, abiraterone and galeterone are indicated.

**Supplementary Fig. 4**

NCI-H295R cells treated with RSL3 and different concentrations of etomidate (A), RSL3 and aminoglutethimide (B), RSL3 and abiraterone (C) and RSL3 and galeterone (D). Note that in NCI-H295R cells 0.5µM RSL3 is close to the EC50 value which explains why cells have sometimes completely died. NCI-H295R treated with RSL3 and incubated with progesterone and pregnenolone (E). HepG2 cells treated with 20 µM RSL3 and either liproxstatin (F) or ferrostatin (G).

**Supplementary Fig. 5**

Inhibition of de-novo cholesterol synthesis by tasin-1 (5µM) did not protect cells from RSL3-induced cell death. By overcoming cholesterol synthesis block by lathosterol supplementation, cell viability was not affected either (A-D).
