# Supplementary figures and images for: Active steroid hormone synthesis renders adrenocortical cells highly susceptible to type II ferroptosis induction
Source: Cell Death Dis. 2020 Mar 17;11(3):192. doi: 10.1038/s41419-020-2385-4 (PMC7078189; doi:10.1038/s41419-020-2385-4)

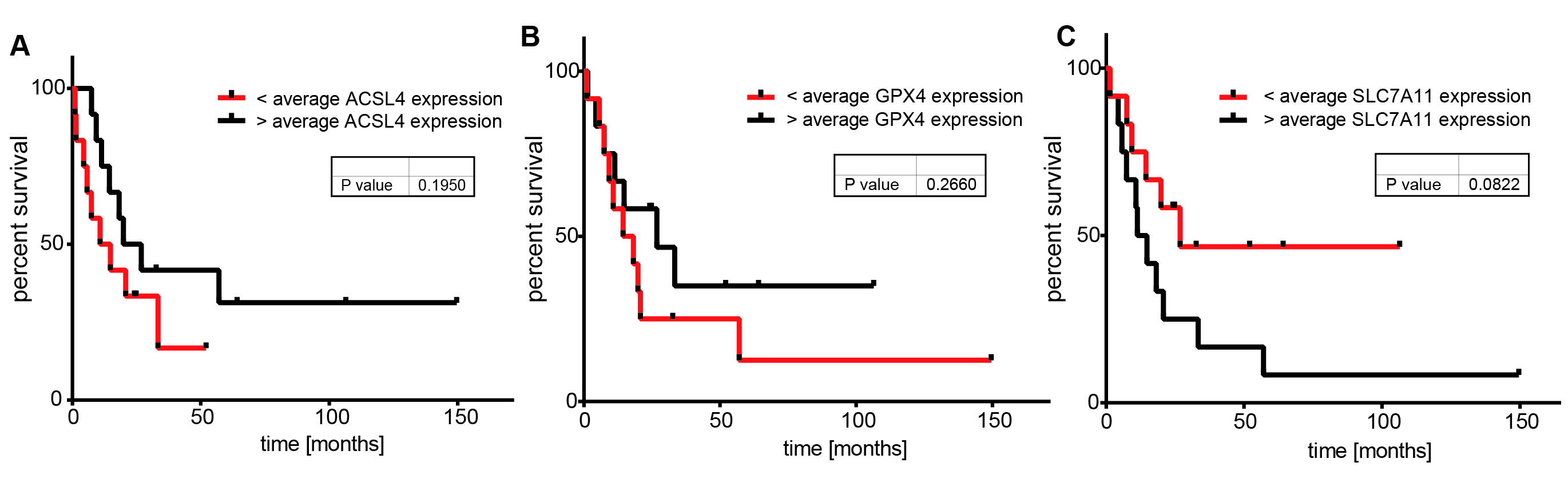

Supplement: Supplementary file 2 — supplementary figure 1 [file 41419_2020_2385_MOESM2_ESM.tif]

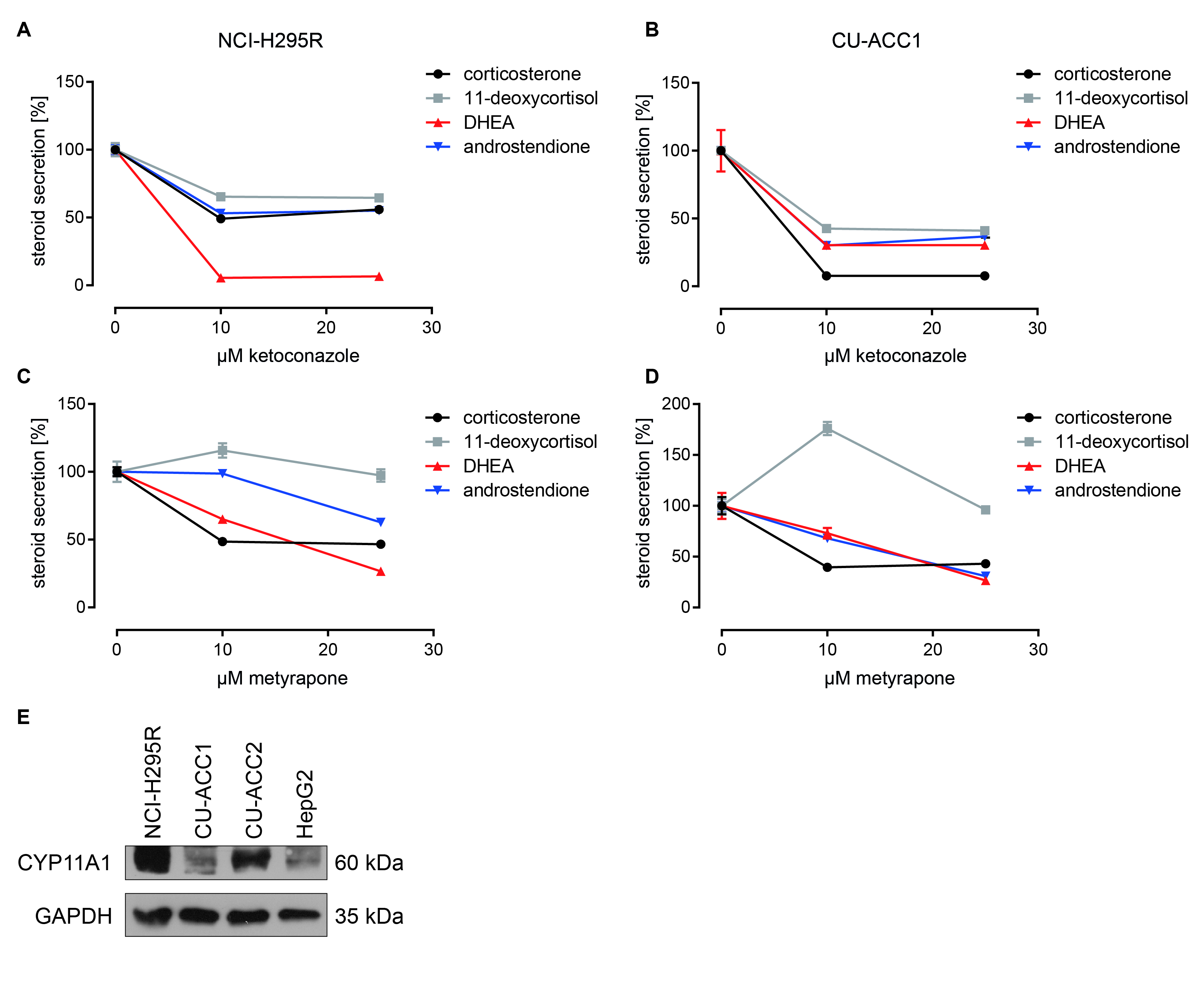

Supplement: Supplementary file 3 — supplementary figure 2 [file 41419_2020_2385_MOESM3_ESM.tif]

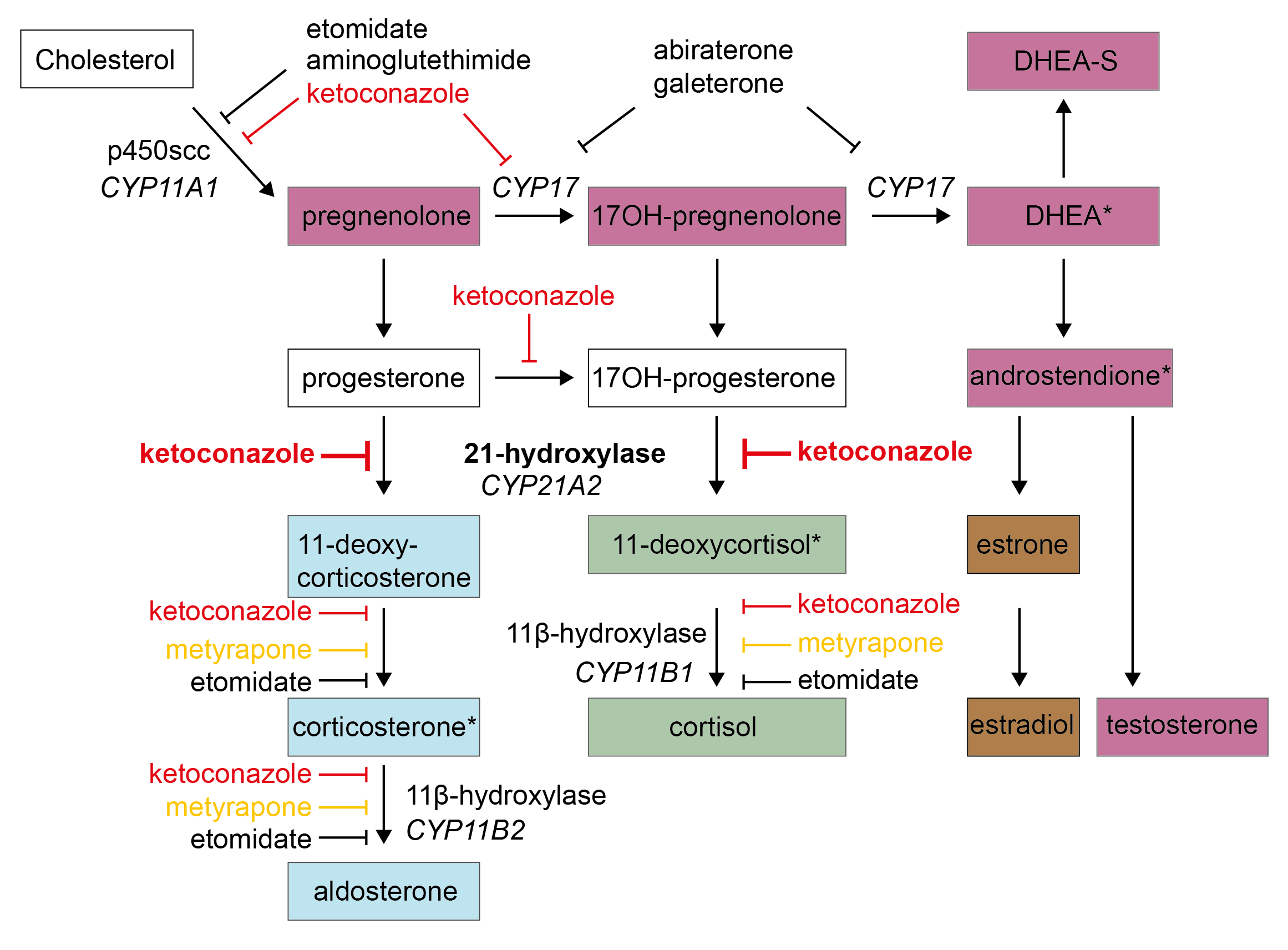

Supplement: Supplementary file 4 — supplementary figure 3 [file 41419_2020_2385_MOESM4_ESM.png]

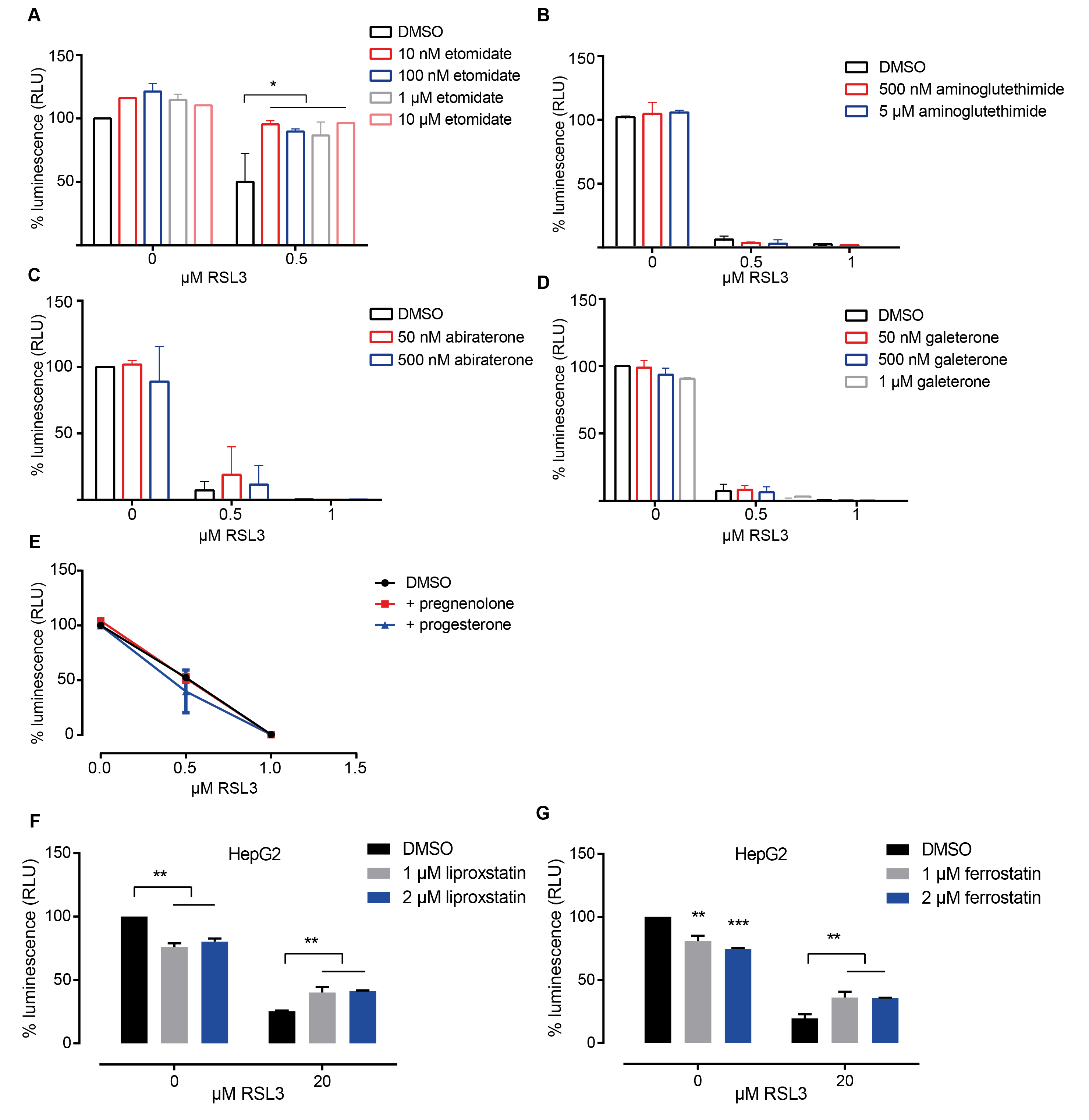

Supplement: Supplementary file 5 — supplementary figure 4 [file 41419_2020_2385_MOESM5_ESM.png]

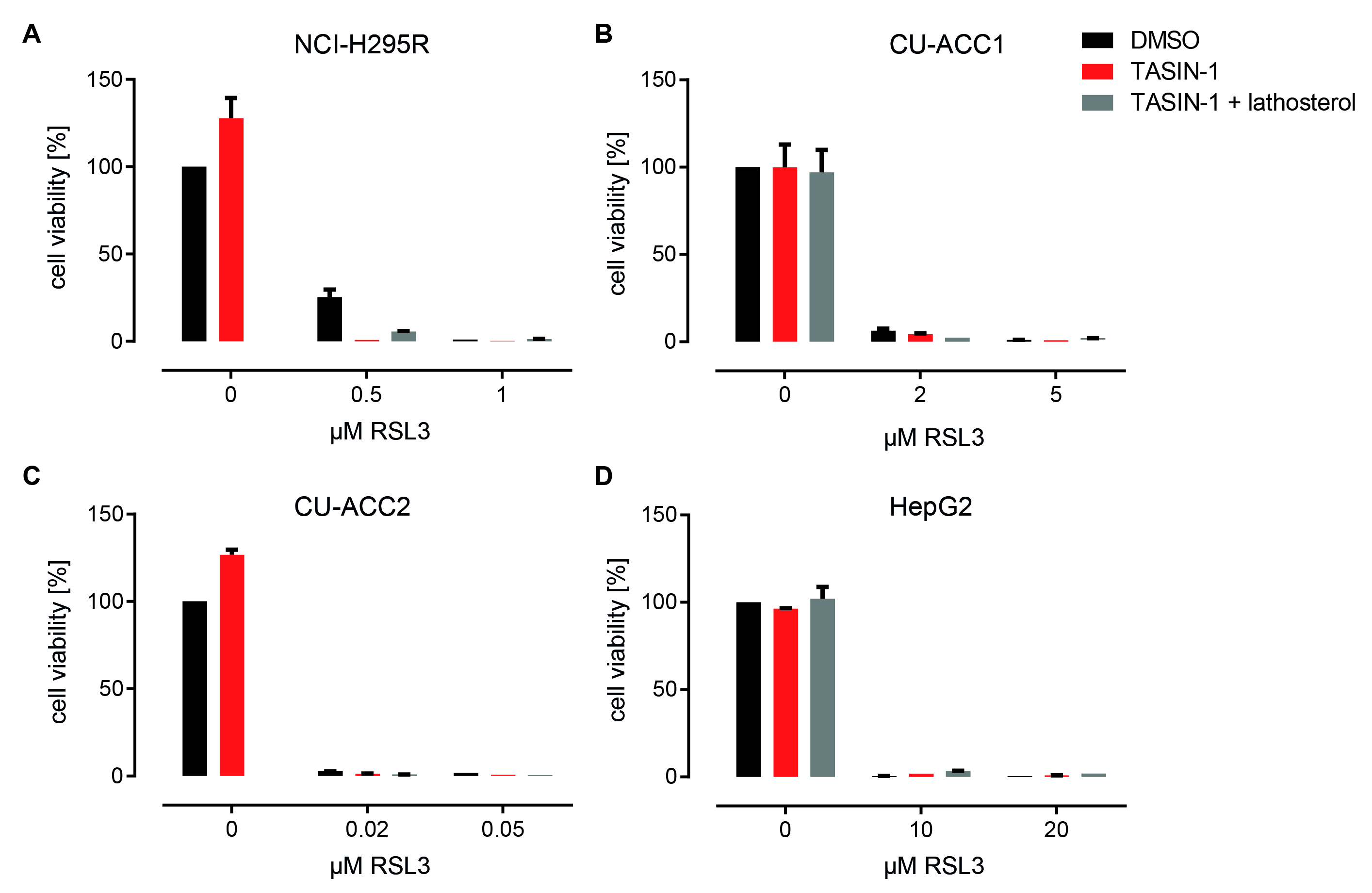

Supplement: Supplementary file 6 — supplementary figure 5 [file 41419_2020_2385_MOESM6_ESM.tif]
